# Clinical, immunological and genetic features in eleven Algerian patients with major histocompatibility complex class II expression deficiency

**DOI:** 10.1186/1710-1492-8-14

**Published:** 2012-08-03

**Authors:** Réda Djidjik, Nesrine Messaoudani, Azzedine Tahiat, Yanis Meddour, Samia Chaib, Aziz Atek, Mohammed Elmokhtar Khiari, Nafissa Keltoum Benhalla, Leila Smati, Abdelatif Bensenouci, Mourad Baghriche, Mohammed Ghaffor

**Affiliations:** 1 Immunology Department, Beni Messous Teaching Hospital, Algiers, Algeria; 2 Immunology Department, Central Hospital of the Army, Algiers, Algeria; 3 Pediatrics Department A, Beni Messous Teaching Hospital, Algiers, Algeria; 4 Pediatrics Department, Bologhine Hospital, Algiers, Algeria; 5 Pediatrics Department B, Beni Messous Teaching Hospital, Algiers, Algeria

## Abstract

Presenting processed antigens to CD4+ lymphocytes during the immune response involves major histocompatibility complex class II molecules. MHC class II genes transcription is regulated by four transcription factors: CIITA, RFXANK, RFX5 and RFXAP. Defects in these factors result in major histocompatibility complex class II expression deficiency, a primary combined immunodeficiency frequent in North Africa. Autosomal recessive mutations in the *RFXANK* gene have been reported as being the principal defect found in North African patients with this disorder. In this paper, we describe clinical, immunological and genetic features of 11 unrelated Algerian patients whose monocytes display a total absence of MHC class II molecules. They shared mainly the same clinical picture which included protracted diarrhoea and respiratory tract recurrent infections. Genetic analysis revealed that 9 of the 11 patients had the same RFXANK founder mutation, a 26 bp deletion (named I5E6-25_I5E6+1, also known as 752delG26). Immunological and genetic findings in our series may facilitate genetic counselling implementation for Algerian consanguineous families. Further studies need to be conducted to determine 752delG26 heterozygous mutation frequency in Algerian population.

## Background

Major histocompatibility complex (MHC) class II deficiency or Bare lymphocyte syndrome (BLS) type II is a primary immunodeficiency, characterized by partial or total absence of constitutive and induced MHC class II molecules (DR, DQ and DP) surface expression on cells normally expressing them [1-3].

Expression of DR, DQ and DP genes is controlled by four transcription factors encoded by four different genes, *CIITA* (MIM 600005), *RFXANK* (MIM 603200), *RFX5* (MIM 601863) and *RFXAP* (MIM 601861) [4-7]. Defects in these factors results in BLS type II and determine four groups of complementation, respectively, A, B, C and D [8-11]. Transcription factors RFXANK, RFX5 and RFXAP associate to form the RFX complex [12] which allow, together with CIITA [7,13,14], CMH class II gene expression. The absence of CMH class II molecules surface expression on antigen-presenting cells is behind impaired immune responses in patients with BLS, who present then, a combined immunodeficiency of early-onset. These individuals are prone to severe and recurrent respiratory and digestive tract infections; whether they are bacterial, viral or fungal. Intestinal infections lead to malabsorption and thereby a failure to thrive. The average life expectancy of these patients is 4 years [15].

A high consanguinity rate has been noted in affected families, whatever the complementation group to which they belong. MHC class II deficiency is inherited as an autosomal recessive trait. Since the first description of this disease, about 150 patients have been reported [16]. Although, this disorder is observed in different ethnic groups, North African population remains the most frequently affected [11,17]. The majority of North African patients belong to complementation group B. These patients have, mostly, the same mutation in the RFXANK gene: a 26 bp deletion at the boundary between intron 5 and exon 6 named I5E6-25_I5E6+1 (also known as 752delG-25) [11] which has been found in more than 90% of North African families [18]. This gene encodes a transcription factor of 260 amino acids. It is composed of ten exons spanning approximately 10 Kb and is located on chromosome 19p12 [5,19].

No genetic study of patients with BLS type II has ever been conducted in Algeria. Here we report clinical and genetic features of eleven Algerian MHC class II deficiency patients, in the light of immunological abnormalities.

## Methods

### Patients

Eleven unrelated Algerian patients with MHC class II deficiency were enrolled among 52 children with primary immune deficiency. They were mainly originated from Kabylie Region (a region in north Algeria populated mostly by aboriginal Berber population). The median age was 3 years and 6 months (range: 2 months–10 years). All our patients being children, consent of participation in this study was obtained from the parents. The regional ethics committee gave approval for the study.

Parents were consanguineous in eight of the eleven families. Ten patients presented a medical history marked by several episodes of diarrhoea and recurrent respiratory tract infections. For genetic analysis, we managed to include both parents for seven of the studied families.

### Immunological analysis

Serum immunoglobulin concentration was determined by nephelometry, using BN ProSpec® System (Siemens). Peripheral blood leucocytes immunophenotyping was carried out by Beckman EPICS ® XL^TM^ flow cytometry, using a panel of monoclonal antibodies directed against CD45, CD3, CD4, CD8, CD19 and HLA DR molecules, labelled with either fluorescein (FITC) or phycoerythrin (PE) (Becton Dickinson). HLA DR expression was assessed on monocytes CD14+.

### DNA extraction and genetic analysis

Genomic DNA from subjects, extracted from whole blood with Qiagen ® kit, was used as template for PCR (Taq PCR Core Kit- Qiagen) using oligonucleotide primers encompassing the intron 5–exon 6 boundary. PCR was performed in a total volume of 50 μl containing 100 ng of template DNA and 10 pmol of each primer (Forward: ggttctctagattggcagcactggggatag, Reverse: gctacgaattccagcagacacagccaaaac) after an initial denaturation step at 94°C for 5 min, 35 cycles of PCR amplification were performed (94°C for 30 s, 60°C for 30 s and 72°C for 45 s each) followed by a final DNA extension at 72°C for 5 min. We screened for the 752delG26 mutation (deletion of 26 bp) by analysing the size of PCR products by electrophoresis in a 2% agarose STG gel: normally, the primers used generate a PCR product of 244 bp. The presence of I5E6-25_I5E6+1 mutation ultimately results in the generation of a 218 bp PCR product. This result was confirmed by DNA sequence Analysis (ABI BigDye Terminator v 3.1 standard kit and an ABI 3130 Genetic Analyzer).

## Results

### Clinical features

Infections were the predominant clinical feature of the disorder. All patients had chronic diarrhoea, except one (patient 4) who was assessed further to a familial history of siblings’ death (two boys). The mean age at first infection was 10 months (range: 2 months- 3 years and 6 months). Six patients had respiratory tract infections (otitis, bronchiolitis and pneumonia). Thrush was found in three patients and molluscum contagiosum in one. Failure to thrive was observed in nine patients. The most severe clinical picture was observed in patient 9, who had protracted diarrhoea, interstitial pneumonia, genito-urinary tract infections, eczema, thrush and multiple abscesses. At the time of this report, all patients were still alive except patient 9 who died following a severe septicaemia.

The median age of MHC class II deficiency diagnosis was 3 years and 6 months (range: 2 months–10 years). Antibiotics prophylaxis was introduced in all patients and IVIG replacement therapy in 8 patients; this allowed a significant decrease in the frequency of infectious episodes. No patient has undergone hematopoietic stem cells transplantation.

### Immunological features

Before introducing immunoglobulin (IgG) substitution therapy, serum immunoglobulin concentrations were heterogeneous: they were low in 8 patients, high in two patients and within normal range in one patient (Table 1). All 11 patients displayed a total absence of MHC class II (HLA-DR) molecule expression on monocytes (CD14+). Low absolute CD4+ T-cell counts were found in nine of the eleven patients; two patients had high and normal levels of T CD4+. TCD8 levels were normal in five patients, high in two patients and low in four patients. Flow cytometry showed, also, normal levels of CD19+ B cells in four patients and decreased in 7 patients (Table 2).

**Table 1:** Serum immunoglobulin concentration in Algerian patients

	**Age at diagnosis M: months; Y: year**	**Serum Ig concentration (g/l)**		
		**IgG**	**IgM**	**IgA**
1	10 y	6.21 (6.55–12.17)	0.34 (0.62–1.77)	0.06 (0.49–1.57)
2	2 y 6 m	4.85 (7.10–10.70)	0.41 (0.48–0.78)	0.24 (0.66–1.20)
3	4 y	4.83 (7–11.60)	1.03 (0.40–0.90)	0.67 (0.79–1.69)
4	2 m	0.93 (2–6)	0.26 (0.12–0.48)	0.25 (0.08–0.34)
5	1 y 10 m	1.63 (5.60–9.60)	0.18 (0.35–0.81)	0.24 (0.26–0.74)
6	4 y	4.77 (7–11.60)	0.73 (0.40–0.90)	0.59 (0.79–1.69)
7	2 y 3 m	10 (5.60–9.60)	1.18 (0.35–0.81)	0.75 (0.26–0.74)
8	4 y	19.10 (7–11.60)	1.42 (0.04–0.90)	2.29 (0.79–1.69)
9	1 y 10 m	6.82 (7–10.60)	0.63 (0.79–1)	1.82 (0.66–1.2)
10	6 y	4.79 (6.8–11.8)	ND	0.07 (0.32–0.98)
11	4 y	4.57 (7–11.60)	0.25 (0.04–0.90)	0.23 (0.79–1.69)

(ND: not determined; normal values for age are given between brackets).

**Table 2:** Immunophenotyping of leucocytes by flow cytometry

**Immunophenotyping of leucocytes (x10**^**3**^**cells/mm**^**3**^**)**							
**Age at diagnosis Y: years/m: months**		**Lymphocytes CD45+**	**CD3+**	**CD3+ CD4+**	**CD3+ CD8+**	**CD19+**	**HLA DR**
1	10 y	2.9 (1.90–3.70)	2.6 (1.20–2.60)	0.6 (0.65–1.50)	1.9 (0.37–1.10)	0.2 (0.27–0.86)	0
2	2 y 6 m	4.6 (2.30–5.40)	2.4 (1.40–3.70)	0.5 (0.70–2.20)	1.5 (0.49–1.30)	1.2 (0.39–1.40)	0
3	4 y	6.2 (2.30–5.40)	4.8 (1.40–3.70)	0.3 (0.70–2.20)	2.9 (0.49–1.30)	0.2 (0.39–1.40)	0
4	2 m	3.3 (3.40–7.60)	1.6 (2.50–5.50)	0.4 (1.60–4.00)	1.2 (0.56–1.70)	0.7 (0.30–2.00)	0
5	1 y 10 m	1.2 (3.60–8.90)	0.3 (2.10–6.20)	0.2 (1.30–3.40)	0.1 (0.62–2.00)	0.4 (0.72–2.60)	0
6	4 y	1.5 (2.30–5.40)	0.7 (1.40–3.70)	0.3 (0.70–2.20)	0.3 (0.49–1.30)	0.3 (0.39–1.40)	0
7	2 y 3 m	4 (2.30–5.40)	2.5 (1.40–3.70)	1.4 (0.70–2.20)	0.6 (0.49–1.30)	1.1 (0.39–1.40)	0
8	4 y	3.7 (2.30–5.40)	2.3 (1.40–3.70)	3.3 (0.70–2.20)	1.1 (0.49–1.30)	0.4 (0.39–1.40)	0
9	2 y	1.2 (2.30–5.40)	0.8 (1.40–3.70)	0.2 (0.70–2.20)	0.5 (0.49–1.30)	0.07 (0.39–1.40)	0
10	6 y	3.2 (1.90–3.70)	2.4 (1.20–2.60)	0.3 (0.65–1.50)	1.5 (0.37–1.10)	0.4 (0.27–0.86)	0
11	4 y	3.5 (2.30–5.40)	1.9 (1.40–3.70)	0.15 (0.70–2.20)	1 (0.49–1.30)	0.18 (0.39–1.40)	0

(Normal values for age are given between brackets) [20].

### Genetic features

**Figure 1:**
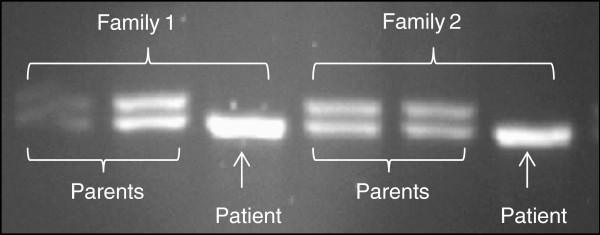
Photograph of a gel showing the 752delG26 deletion of the RFXANK gene: patients are homozygous; parents are heterozygous for the deletion.

Size analysis of PCR products encompassing the boundary between intron 5 and exon 6 for all patients allowed the detection of 752delG26 mutation (deletion of 26 bp) in 9 patients in the homozygous state (a single 218 bp band observed in the agarose gel). All the parents tested presented this mutation in the heterozygous state (two bands of 244 bp and 218 bp in size) (Figure 1). Consanguinity was strongly incriminated in this immunodeficiency in our series, since eight of eleven patients were descended from consanguineous parents.

## Discussion

Major histocompatibility complex type II is a rare immunodeficiency with autosomal recessive transmission [3,18,21]. It is due to mutations in four MHC II regulatory genes, thus defining four complementation groups: *MHC2A* for group A, *RFXANK* for group B, *RFX5* for group C and *RFXAP* for group D [8,9,11]. This disorder is particularly frequent in North Africa [11,20], where a high rate of consanguinity is found [22-25]. It is characterized by the occurrence of chronic diarrhoea and recurrent respiratory tract infections. First clinical manifestations are of early onset, generally within the first year of life [15,26]. In our series, the median age was 10 months. Death at an early age was reported for one or several of the siblings of some patients. Symptoms in our patients were similar to those reported in other series of MHC class II deficiency [15,16,27,28]. All the patients lacked HLA DR surface expression on monocytes; however, we observed variability in immunological phenotype [15], which may be either the result of environmental influences or could reflect the existence of additional genetic factors. Although, two patients had normal and high levels of TCD4+, these findings do not exclude the diagnosis (Table 3).

**Table 3:** Clinical and genetic features of the 11 Algerian patients with MHC class II deficiency

**Patients**	**Sex**	**Age at diagnosis**	**Consanguinity**	**Death in siblings (with similar symptoms)**	**Morbidities**	**I5E6-25_I5E6+1 mutation**
1	F	10 y	+	None	Chronic diarrhoea, respiratory tract infections	+
2	M	2 y 6 m	+	None	Chronic diarrhoea	+
3	M	4 y	+	2 brothers	Chronic diarrhoea, thrush	+
4	F	2 m	+	2 brothers	None	+
5	F	1 y 10 m	+	1brother, 1 sister	Chronic diarrhoea, thrush	+
6	M	4 y	+	None	Chronic diarrhoea, respiratory tract infections	+
7	M	2 y 3 m	+	None	Chronic diarrhoea, respiratory tract infections	−
8	F	4 y	−	None	Chronic diarrhoea, respiratory tract infections, molluscum contagiosum	+
9	M	1 y 10 m	−	None	Chronic diarrhoea, respiratory tract infections, abcess, eczema, thrush, genito-urinary tract infections	−
10	M	6 y	−	None	Respiratory tract infections	+
11	F	4 y	+	None	Chronic diarrhoea, respiratory tract infections	+

Genetic analysis showed that nine patients carried the 752delG26 mutation at the homozygous state in the RFXANK gene, which is present in 92% of affected North African patients. This mutation has been identified as the most frequent molecular defect in patients belonging to complementation group B. Nevertheless, Eight other mutations in RFXANK have been described in patients of different ethnic origins (Netherlands, Italy, France/Spain, Tunisia, Turkey, Saudi Arabia) [5,29-33]; the two remaining patients (patients 7 and 9) are being tested for these mutations. It has been suggested that I5E6-25_I5E6+1 mutation originated from a Maghrebian ancestor [16,33]. This defect results in a frame shift and the introduction of a premature stop codon in exon 9. In our series, 9 patients carried this mutation which is consistent with the literature data concerning the high frequency of I5E6-25_I5E6+1 mutation in Algerian and Maghrebian patients [33,34]. Recently, this mutation have been dated in a series of 35 MHC class II deficiency patients; according to this study, the founder event responsible for this mutation in this population have occurred about 2,250 years ago [16]. Ten patients remain alive while one patient (patient 9) died further to severe septicaemia. Interestingly, two patients (1 and 10) survived beyond the expected life span. For patient 1, this may be explained by a delay in the referral of the patient to our center; as for patient 10, the delay in diagnosis is due to a milder onset of the disease. Adult patients with MHC class II expression deficiency have been reported in some studies [16,35]. The reasons for this variability in immunological phenotype remain unknown.

Although HSCT is the only known curative treatment for MHC class II expression deficiency, none of the patients has undergone hematopoietic stem cells transplantation. HSCT is strongly related with the age and the infectious status, and has limited success rate in patients with infections [16]. Better results have recently been obtained for HSCT with grafts from unrelated donors [16].

Immunological and genetic findings in our series demonstrate that the most frequent mutation found in Algerian patients, I5E6-25_I5E6+1, has a possible founder effect, as previously suggested by Wiszniewski and al. This may facilitate genetic counselling implementation for Algerian consanguineous families. Further studies need to be conducted to determine the I5E6-25_I5E6+1 heterozygous mutation frequency in Algerian population and the exact impact of epigenetic and environmental factors on MHC class II deficiency phenotype.

## Competing interests

The authors report no conflict of interests.

## Authors’ contributions

RD and NM: conceived the study, participated in its design and coordination and wrote the manuscript. YM and SC: carried out the molecular genetic studies. AT: carried out the immunoassays. NKB, LS, AA, MEK, AB and MB: participated in the design of the study. MG: conceived the study, participated in its design and coordination and helped to draft the manuscript. All authors read and approved the final manuscript.
